# CD147 mediates epidermal malignant transformation through the RSK2/AP-1 pathway

**DOI:** 10.1186/s13046-022-02427-w

**Published:** 2022-08-13

**Authors:** Xu Zhang, Yeye Guo, Ta Xiao, Jie Li, Aiyuan Guo, Li Lei, Chong Jin, Qi Long, Juan Su, Mingzhu Yin, Hong Liu, Chao Chen, Zhe Zhou, Susi Zhu, Juan Tao, Shuo Hu, Xiang Chen, Cong Peng

**Affiliations:** 1 Department of Dermatology, Xiangya Hospital, Central South University; Hunan Key Laboratory of Skin Cancer and Psoriasis and Hunan Engineering Research Center of Skin Health and Disease; National Clinical Research Center for Geriatric Disorders (Xiangya Hospital), Xiangya Road #87, Changsha, 410008 Hunan China; 2 National Engineering Research Center of Personalized Diagnostic and Therapeutic Technology, Changsha, 41008 China; 3 Institute of Dermatology, Chinese Academy of Medical Science & Peking Union Medical College, Nanjing, 210042 China; 4 Department of Dermatology, The 3rd Xiangya Hospital, Central South University, Changsha, 410000 China; 5 Department of Biostatistics, Epidemiology and Informatics, University of Pennsylvania, Philadelphia, PA 19104 USA; 6 Department of Dermatology, Affiliated Union Hospital, Tongji Medical College, Huazhong University of Science and Technology, Wuhan, 430022 China; 7 Department of Nuclear Medicine, XiangYa Hospital, Central South University; Key Laboratory of Biological Nanotechnology of National Health Commission, Changsha, 410000 Hunan China

**Keywords:** CD147, Keratinocyte, Malignant transformation, RSK2

## Abstract

**Background:**

Malignant transformation of the epidermis is an essential process in the pathogenesis of cutaneous squamous-cell carcinoma (cSCC). Although evidence has demonstrated that CD147 plays key roles in various tumors, the role of CD147 in epidermal malignant transformation in vivo remains unclear.

**Methods:**

Epidermal CD147-overexpression or knockout (Epi^CD147-OE^ or Epi^CD147-KO^) transgenic mouse models were generated for in vivo study. RNA-sequencing and q-PCR were performed to identify the differentially expressed genes. Immunohistochemistry and flow cytometry were performed to investigate the role of CD147 in regulating myeloid-derived suppressor cells (MDSCs). Immunoprecipitation, EMSA and ChIP assays were performed to investigate the mechanism of CD147 in cell transformation.

**Results:**

We found that specific overexpression of CD147 in the epidermis (Epi^CD147-OE^) induces spontaneous tumor formation; moreover, a set of chemokines and cytokines including CXCL1, which play essential function in MDSC recruitment, were significantly upregulated in Epi^CD147-OE^ transgenic mice. As expected, overexpression of CD147 in the epidermis remarkably facilitated tumorigenesis by increasing the rate of tumor initiation and the number and size of tumors in the DMBA/TPA mouse model. Interestingly, the expression of CXCL1 and the infiltration of MDSCs were dramatically increased in Epi^CD147-OE^ transgenic mice. Our findings also showed that knockdown of CD147 attenuated EGF-induced malignant transformation as well as CXCL1 expression in HaCaT cells. Consistently, CD147 was found overexpressed in cutaneous squamous cell carcinoma (cSCC), and positively related with the expression of CD33, a myeloid-associated marker. We further identified RSK2, a serine/threonine kinase, as an interacting partner of CD147 at the binding site of CD147^D207-230^. The interaction of CD147 and RSK2 activated RSK2, thus enhancing AP-1 transcriptional activation. Furthermore, EMSAs and ChIP assays showed that AP-1 could associate with the CXCL1 promoter. Importantly, RSK2 inhibitor suppressed the tumor growth in DMBA/TPA mouse model by inhibiting the recruitment of MDSCs.

**Conclusion:**

Our findings demonstrate that CD147 exerts a key function in epidermal malignant transformation in vivo by activating keratinocytes and recruiting MDSCs via the RSK2/AP-1 pathway.

**Supplementary Information:**

The online version contains supplementary material available at 10.1186/s13046-022-02427-w.

## Background

Epidermal tumorigenesis is a multistage process involving a variety of genetic or epigenetic alterations [1]. Chemical carcinogens, such as 7,12-dimethylbenzanthracene/12-O-tetradecanoylphorbol-13-acetate (DMBA/TPA), can be administered to induce mouse skin carcinogenesis, providing a model for investigating the biological processes involved in tumor pathogenesis [2]. In this process, normal keratinocytes gain malignant behaviors, including anchorage-independent growth, enhanced migration and invasion, which is a key step in tumorigenesis. Various molecules, such as PI3K/Akt, Wnt/β-catenin [3], NF-κB [4], and AP-1, have been validated to contribute to malignant transformation. AP-1 is well known to be an essential transcriptional factor for the regulation of cell growth, differentiation and transformation, and activation of AP-1 has been documented in the conversion of normal keratinocytes to carcinoma cells [5, 6].

The relationship between chronic inflammation and tumor development is well documented [7]. The secretion of inflammatory factors (cytokines or chemokines) by tumor cells or the infiltration of inflammatory cells, including myeloid-derived suppressor cells (MDSCs), create a tumor microenvironment (TME) favorable for tumor development [8]. MDSCs are a heterogeneous population of immature myeloid cells, including precursors for granulocytes, macrophages and dendritic cells (DCs), which have been recruited to specific tissues or organs through chemokines such as IL-6, CXCL1, and CXCL2 under pathological conditions such as tumorigenesis and chronic inflammation [9, 10]. MDSCs can generate an immunosuppressive environment through secreting nitric oxide (NO), arginase, cytokines or reactive oxygen species (ROS) that suppress CD4^+^ or CD8^+^ cell function [11].

CD147, also called Basigin or EMMPRIN, is a transmembrane glycoprotein belonging to the immunoglobulin superfamily [12]. Studies have demonstrated that CD147 is highly expressed in various cancers [13–15]. Our previous work demonstrated that CD147 promotes cutaneous melanoma by interacting with chaperones, such as MMPs [16], GLUT-1 [17] and TRAF6 [18], and enhances the cSCC malignant phenotype by regulating EGFR [19]. However, there is a lack of in vivo experimental evidence supporting the crucial role of CD147 in tumorigenesis. Therefore, we generated transgenic mice with epidermal overexpressing (Epi^CD147-OE^) or knockout (KRT14Cre^+^/CD147^fl/fl^ Epi^CD147-KO^) of CD147 for further investigation. Surprisingly, we noticed that tumors spontaneously developed in Epi^CD147-OE^ mice; furthermore, assessment of the DMBA/TPA-induced skin cancer model validated the role of CD147 in malignant transformation and carcinogenesis, suggesting that CD147 has the ability to transform normal keratinocytes and initiate tumorigenesis.

## Materials and Methods

### Cell lines and cell culture

The human keratinocyte cell line HaCaT, murine keratinocyte cell line JB6 Cl 41-5a, human squamous cell carcinoma cell line A-431 and HEK293T were maintained in Dulbecco’s modified Eagle’s medium (DMEM, 01-052-1A, VivaCell, Shanghai, China) with 10% fetal bovine serum (04-001-1A, VivaCell, Shanghai, China) and 1% antibiotics (10,000 μg/ml streptomycin and 10,000 units/ml penicillin, 03-031-1B, Biological Industries, Israel) at 37 °C and 5% CO2.

### Antibodies and reagents

Chemical reagents, including Tris base, glycine, NaCl, and SDS for molecular biology and buffer preparation, were purchased from Sigma-Aldrich (St. Louis, MO, USA). The primary antibodies used were mouse monoclonal anti-CD147 antibody (1:1000, ab666, Abcam, MA, USA), rabbit monoclonal anti-phospho-RSK2 (Ser227) antibody (1:1000, 3556 s, Cell Signaling Technology, MA, USA), rabbit monoclonal anti-RSK2 antibody (1:1000, 5528 s, Cell Signaling Technology, MA, USA), mouse monoclonal anti-c-fos antibody (1:1000, 66590, proteintech, IL, USA), mouse monoclonal anti-CD33 antibody (abcam, ab11032, MA, USA) and mouse monoclonal anti-GAPDH antibody (1:3000, 60,004-1-Ig, Proteintech, IL, USA).

### Transgenic mouse model

Xenograft tumor models were constructed by Shanghai Model Organisms (Shanghai, China). Briefly, for CD147 epidermal overexpressing (Epi^CD147-OE^) mice, CD147 genomic DNA was inserted into pCAG-pcDNA3.1 (pCAG-CD147), followed by digestion with *pvul*. The 5.3-kb fragment was isolated and purified before microinjection into fertilized one-cell stage mouse oocytes. Transgenic mice were generated from C57BL/6 J embryos by standard microinjection procedures. The transgenic line Epi^CD147-OE^ was established by breeding heterozygous mice with C57BL/6 J mice. Littermates were used as controls. For CD147 epidermal knockout transgenic mice (KRT14 Cre^+^/CD147^*fl/fl*^, Epi^CD147-KO^), a donor vector including a 4.5-kb 5′ arm of homology, 2.0-kb loxP and a 4.6-kb 3′ arm of homology was constructed by in-fusion cloning. Cas9 mRNA, gRNA and donor vector were microinjected into fertilized C57BL/6 J oocytes to obtain CD147^*fl/+*^ mice. Chimeras were then mated to KRT14 Cre^+^ mice to generate KRT14 Cre^+^/CD147^*fl/+*^ or KRT14 Cre^−^/CD147^*fl/+*^. KRT14 Cre^+^/CD147^*fl/+*^ and CD147^*fl/+*^ mice were mated to generate KRT14 Cre^+^/CD147^*fl/fl*^ and KRT14 Cre^−^/CD147^*fl/lf*^, respectively. To identify transgenic animals, DNA was extracted from the tail biopsies of 3-week-old mice and analyzed by PCR with probes and primers (P1 and P2 for Epi^CD147-OE^, P3 and P4 for Epi^CD147-KO^) designed to discriminate between human and murine genomic CD147 DNA, as shown below.

*P1: 5’CGATCACGAGACTAGCCTC’3.*

*P2: 5’GTCATCTGCGTCCACTATGT’3.*

*P3: 5’AGCGATGGATTTCCGTCTCTGG’3.*

*P4: 5’AGCTTGCATGATCTCCGGTATTGAA’3.*

### DMBA/TPA skin carcinogenesis model

The backs of the mice were shaved 2 days prior to the start of the treatment, and then the mice received 250 μg/mL DMBA in acetone (200 μl/mouse) for 2 weeks, followed by 50 μg/mL TPA in acetone (50 μl/mouse) every 3 days for 24 weeks. The appearance of papillomas > 2 mm in diameter was recorded every week from the first appearance at 8 weeks. Tumors were measured by calipers, and tumor volume was calculated using the following formula: length × width × height × 0.52. After 24 weeks, the mice were sacrificed, and the tumor tissues were harvested and fixed in 10% buffered formalin, embedded in paraffin, sectioned at a thickness of 5 μm, and stained with H&E or subjected to immunohistochemical analysis.

### Flow cytometry

The skin lesions of mice were digested with collagenase IV (2 mg/mL) and DNAse I (50 U/ml) for 2 hours. Then, the cell suspensions were blocked and stained with reagents from the Live/Dead Fixable Aqua Dead Cell Stain Kit (Life Technologies) to distinguish between live and dead cells. Then cells were labeled with indicated antibodies, including CD45 (103116, Biolegend), Gr-1 (108428, Biolegend), CD11b (101208, Biolegend) for 30 min on ice. Samples were then analyzed on flow cytometer.

### Protein preparation and Immunoblotting

Cells were lysed in RIPA buffer, and protein concentrations were determined by a BCA Protein Assay Kit (Santa Cruz, CA, USA). Membrane protein was purified by Mem-PER™ Plus Membrane Protein Extraction Kit (Thermo Scientific™, MA, USA). A total of 30-50 μg of protein was separated by sodium dodecyl sulfate (SDS)-polyacrylamide gel electrophoresis (PAGE) and electroblotted onto polyvinylidene fluoride membranes (Millipore, Billerica, MA). Immunoreactions were detected by an imaging system (Bio-Rad, USA).

### Electrophoretic mobility shift assay

Nuclear protein was extracted by NE-PER™ Nuclear and Cytoplasmic Extraction Reagents (78835, Thermo Scientific™, MA, USA) under the guidance of the manufacturer’s instructions. Three microgram of nuclear protein was subjected to AP-1 kit (AP-1 IRDye 700, 829-07925, LI-COR Biosciences, USA) according to the manufacturer’s instructions.

### Cell proliferation assays

Cell viability was assessed using a Cell Counting Kit-8 (CCK-8) assay (Bimake, USA) according to the manufacturer’s instructions. Cells were seeded into 96-well plates at 3000 cells/well and cultured for 24, 48, 72, or 96 h. Then, 10 μL of CCK-8 solution was added to each well, and the 96-well plate was incubated for 2 h at 37 °C and 5% CO2. The fluorescence of each plate was measured using a spectrophotometer at an emission wavelength of 450 nm (Beckman, USA). Six replicates per sample were analyzed.

### Plasmid and Lentiviral vector construction

Lentivirus plasmids including pLKO.1, pSPAX2, pMD2G and CD147 shRNAs were purchased from Thermo Scientific, MA, USA. The pLVX-CD147-puro (CD147-overexpressing lentivirus plasmid) was constructed in our lab. For the transfection experiments, cells were transfected with different plasmids using TurboFect Transfection Reagent (Thermo Scientific, MA, USA). The reagent and DNA were diluted in DMEM and incubated for 20 min. The mixture was added to cells growing in the plates for 36 to 48 h to facilitate transfection. To establish stable CD147 knockdown and overexpression cells, pLKO.1-shCD147 or pLKO.1-shMock and pLVX-CD147-Puro or pLVX-IRES-Puro plasmids were cotransfected with packaging plasmids (pSPAX2 and PMD2G) into 293 T cells. The supernatant fractions containing lentiviral particles were collected separately at 48 and 72 h, and JB6, HaCaT and A431 cells were infected with lentiviral particles in medium supplemented with 10 μg/mL polybrene. At 16 h after infection, the medium was replaced with fresh medium containing a suitable concentration of puromycin. The appropriate experiments were performed with these cells until all control cells (uninfected) were dead (usually 36-48 h) in the puromycin-containing medium.

### Transwell invasion assay

For the invasion assay, a Transwell experiment was performed with 8 μm pore chambers inserted into 24-well plates (Corning, NY, USA). Matrigel (BD Biosciences, NJ) was diluted (1:7) in serum-free DMEM and was then added to each chamber and allowed to solidify completely. Transfected cells were obtained, resuspended in serum-free medium at a concentration of 4 × 10^4^/100 μL and seeded in the upper chambers, while 550 μL of DMEM containing 30% FBS was placed into the bottom chamber as a chemotactic factor. After 24 or 48 h, cells were fixed in 4% paraformaldehyde for 15 min at room temperature. Nonmotile or noninvaded cells on the top surface of the filter were removed, while motile or invaded cells on the bottom surface were stained with crystal violet. ImageJ software was used to quantify the invaded and migrated cells. Three fields per well were counted with an inverted microscope system (Ti-S, Nikon, Tokyo, Japan).

### Scratch assay

Cells in complete medium were seeded in a 6-well plate at a density of 1 × 10^5^ cells/well, and a straight line was scratched on the cell monolayer with a 200 μL pipette tip. Then, cells were washed with PBS three times to remove debris and then replenished with fresh medium.

### Immunohistochemistry

Formalin-fixed, paraffin-embedded tumor sections were baked at 65 °C, deparaffinized in turpentine, rehydrated through a series of graded alcohols, and immersed in hydrogen peroxide to block endogenous peroxidase activity. Antigen retrieval was performed by heat treatment in a pressure cooker in citrate buffer (pH 6.0) for 3 min. Primary antibodies are as follows: CD147 (1:400, ab666, Abcam, MA, USA) and PCNA (1:300, YM6090, ImmunoWay Biotechnology Company, TX, USA). Sections for immunohistochemistry staining were blocked for nonspecific binding by incubation in normal goat serum at room temperature. Subsequently, slides were incubated with a primary antibody at 4 °C overnight. The next day, sections were incubated with a biotin-conjugated secondary antibody for 20 min and then with peroxidase-conjugated streptavidin for an additional 30 min. Next, 3,3′-diaminobenzidine tetrahydrochloride was used to visualize the reaction, and slides were then counterstained with hematoxylin.

### RNA-sequencing (RNA-Seq)

The cDNA library construction, library purification and transcriptome sequencing were implemented according to the Wuhan Huada Sequencing Company’s instructions (www.genomics.org.cn, BGI, Shenzhen, China).

### Quantitative real-time PCR analysis

Total RNA was extracted from skin lesions of transgenic mice and cells with Trizol reagent. Total RNA (3 μg) was used as the template for the reverse transcription reaction (SuperScript III First-Strand Synthesis System for Reverse Transcription PCR, Invitrogen). All PCR primers used in this study are as follows:

**Table:** 

*CXCL1*	Forward	*5’TGCGCTGCCAGTGCTTGC’*
	Reverse	*5’TTCCGCCCATTCTTGAGTGTGG’*
*CXCL2*	Forward	*5’CACTGGTCCTGCTGCTGCTG’*
	Reverse	*5’GGCGTCACACTCAAGCTCTGG’*
*CXCL10*	Forward	*5’TGCCTCATCCTGCTGGGTCTG’*
	Reverse	*5’CATTCTCACTGGCCCGTCATCG’*
*CCR1*	Forward	*5’AAGGTCAAAGCCGTGCGTCTG’*
	Reverse	*5’GGCCAGGTCCAGTTGCTTACTC’*
*CCL2*	Forward	*5’ACGCCCCACTCACCTGCTG’*
	Reverse	*5’CCTGCTGCTGGTGATCCTCTTG’*
*CCL3*	Forward	*5’CCACCACTGCCCTTGCTGTTC’*
	Reverse	*5’GCGTGGAATCTTCCGGCTGTAG’*
*CCL4*	Forward	*5’CTTGCTCGTGGCTGCCTTCTG’*
	Reverse	*5’AGCTGCCGGGAGGTGTAAGAG’*
*IL-1β*	Forward	*5’GCAGCAGCACATCAACAAGAGC’*
	Reverse	*5’AGGTCCACGGGAAAGACACAGG’*
*MMP8*	Forward	*5’CACGTCTGGAGTGTAGCATCGC’*
	Reverse	*5’TGGTTGAAAGGCATGGGCAAGG’*
*GAPDH*	Forward	*5’GAGTGGGTGTCGCTGTTGAAGTC’*
	Reverse	*5’GGCAAATTCAACGGCACAGTCAAG’*

The qRT-PCR assays and data collection were performed on a 7500 real-time PCR system (Applied Biosystems, USA). The data were analyzed by using the 2^-△△CT^ values.

### ELISA assays

ELISA was used to detect the levels of CXCL1 and CXCL2. ELISA kit (ml037774, ml058180, Mlbio, Shanghai, China) were used. All operations were performed in strict accordance with the manufacturers instructions.

### Luciferase reporter gene assays

pGL3 is the luciferase reporter vector (E1751, Promega, USA). pENTER is the vector with kanamycin selection and CMV promoter and C-terminal FLAG and His tags (pENTER, P100001, Vigene, USA). pRLTK is renilla luciferase control reporter vectors (P100001, Promega, USA). HEK293T cells were transfected with CD147, PGL3-AP-1 and pRLTK. 293 T cells were transfected with CD147 or pEnter, pGL3-AP-1 or pGL3-ctrl and pRLTK pasmids. After 24 h of transfection, the firefly and renilla luciferase activity in the cell lysates was analyzed with a dual luciferase assay kit (Promega, Madison, WI) following the protocol. For each transfection, the luciferase activity of four replicates was averaged.

### Chromatin Immunoprecipitation

HaCaT cells (10^^7^) infected with sh-Mock and sh-CD147 were collected and Chromatin Immunoprecipitation were performed according to the protocol provided by EZ CHIP KIT (Millipore, 17-371RF, MA, USA). Soluble lysates were rotated with 5 μl antibody (c-Jun, Cell Signaling Technology, 9165 s, MA, USA) overnight at 4 °C with protease inhibitors. CXCL1 promoter regions were amplified by PCR (50 cycles) using the following primer pairs:

**Table:** 

*CXCL1-Primer-1*	Forward	*5’AGAGTTCATGTTATACAG’*
	Reverse	*5’TAACATCAGTGCTGTCTG’*
*CXCL1-Primer-2*	Forward	*5’GAAGGGTGCTTGCACACC’*
	Reverse	*5’GGGGTTCAGGTTTGATC’*
*CXCL1-Primer-3*	Forward	*5’TCTCTAGAACTTCTGGAG’*
	Reverse	*5’GCAGACATGCATTCGGATG’*
*CXCL1-Primer-4*	Forward	*5’CAAACATTTATAAGGCAC’*
	Reverse	*5’GAACACAGGCAAGGCTGC’*
*CXCL1-Primer-5*	Forward	*5’GACTGGAGTACATATACTATC’*
	Reverse	*5’TCGGTTACTCAGGGGTAC’*

### Statistical analysis methods

Statistical results are presented as the means ± S.D.s and were analyzed by Student’s t-test or one-way ANOVA to evaluate the statistical differences. A *p*-value of < 0.05 was considered statistically significant.

## Results

### Specific overexpression of CD147 in the epidermis leads to spontaneous tumor formation

**Fig. 1:**
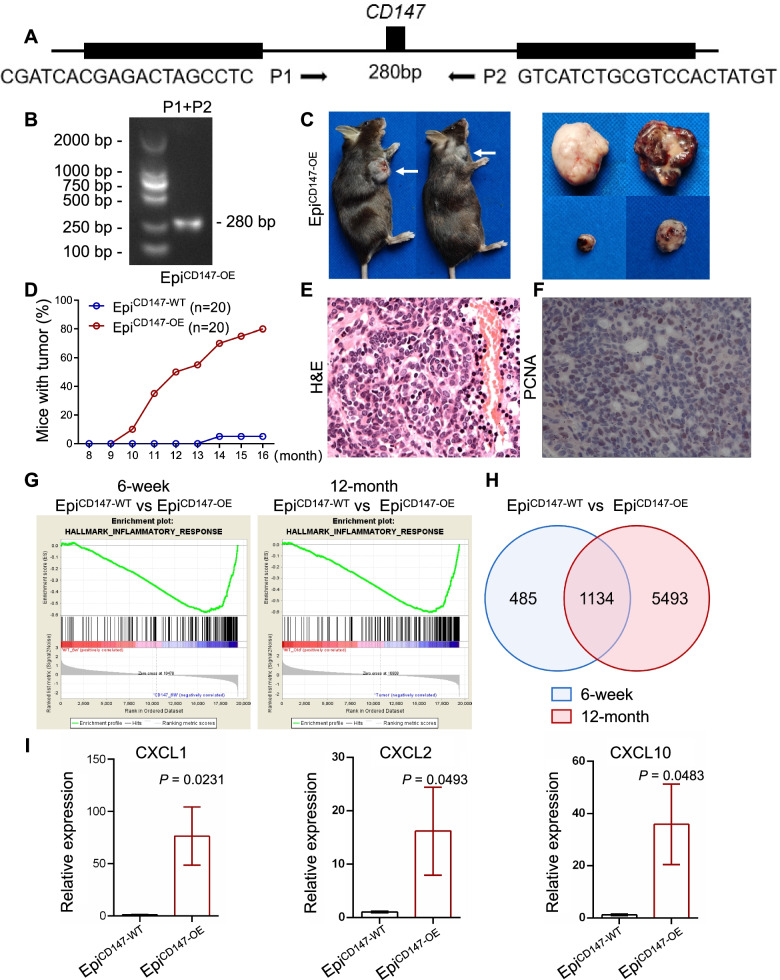
Overexpression of CD147 in the epidermis results in spontaneous tumors formation in transgenic mice and affect the gene expression profile. **A-B** Schematic diagram of primers for genotyping and targeting strategies. Epi^CD147-OE^ genotyping was carried out with Primer 1 (P1) and Primer 2 (P2). **A**, The 280-bp band was obtained for Epi^CD147-OE^ genotyping (**B**), and DNA samples were prepared from total skin. **C-F** Epi^CD147-OE^ mice grew tumors spontaneously. **C** Representative images of tumors from Epi^CD147-OE^ mice (Scale bar = 1 cm). **D** The tumor formation rate of transgenic mice was recorded. Seventy percent (14/20) of Epi^CD147-OE^ mice and 5% (1/20) of Epi^CD147-WT^ mice grew one or more tumors by the endpoint of the 16-month observation. Hematoxylin & eosin staining (**E**) and immunohistochemistry staining of PCNA (**F**) were performed as described in the *Materials and Methods*. **G** The inflammatory response is elevated in Epi^CD147-OE^ mice. Skin tissues from the same part of 6-week and 12-month Epi^CD147-OE^ mice and Epi^CD147-WT^ mice were collected. RNA-seq was performed as described in the *Materials and Methods*, and GSEA of Epi^CD147-WT^ and Epi^CD147-OE^ mice at 6 weeks (left panel) or 12 months of age (right panel) was performed. **H** Venn diagram of differentially expressed genes between 12-month Epi^CD147-OE^ mice vs 12-month Epi^CD147-WT^ mice and 6-week Epi^CD147-OE^ mice vs 6-week Epi^CD147-WT^ mice. Differentially expressed genes were analyzed using DESeq2. **I** CXCLs were elevated in Epi^CD147-OE^ mice. RNA was extracted from Epi^CD147-OE^ and Epi^CD147-WT^ mice. RT-PCR was then performed with different primers for CXCL1, CXCL2 and CXCL10 as described in the *Materials and Methods*. Data from multiple experiments (*n* = 4) are expressed as the mean ± SD. Significant differences were evaluated using one-way Student’s t-test

Although CD147 plays a variety of roles in different tumors, the role of CD147 in epidermal malignant transformation in vivo is unclear; therefore, we generated Epi^CD147-OE^ (Fig. 1A-B). Interestingly, we found that as time passed, tumors spontaneously developed in Epi^CD147-OE^ mice; the tumor formation rate was approximately 55% (11/20) at 12 months and 80% (16/20) at 16 months, while only 5% of control mice (1/20) formed tumors spontaneously (Fig. 1C-D). Hematoxylin and eosin (H&E) staining showed that atypia of the tumor cells from Epi^CD147-OE^ mice (Fig. 1E), with a 16.45 ~ 26.23% positive PCNA staining rate, indicating cell proliferation (Fig. 1F).

### Effect of CD147 on the gene expression profiles of transgenic mice

To investigate the molecular mechanism of CD147 in tumorigenesis, RNA sequencing was conducted to examine the differences in the gene expression profiles of 6-week-old and 12-month-old Epi^CD147-OE^ mice and control mice. We found that the expression of components of the inflammatory response pathway were significantly increased in both 6-week and 12-month Epi^CD147-OE^ mice (Fig. 1G) by gene set enrichment analysis (GSEA). Moreover, 1134 differentially expressed genes were overlapped between the 12-month Epi^CD147-OE^ mice vs 12-month Epi^CD147-WT^ mice and 6-week Epi^CD147-OE^ mice vs 6-week Epi^CD147-WT^ mice comparisons (Fig. 1H). Kyoto Encyclopedia of Genes and Genomes (KEGG) analysis revealed that those differentially expressed genes were also involved in inflammatory signaling pathways, such as the cytokine-cytokine receptor interaction pathways (Supplementary Fig. 1A). We generated a protein-protein interaction (PPI) network and identified that chemokines or cytokines, including CXCLs and CCRs acted as key nodes in this network (Supplementary Fig. 1B). Then, we verified that differentially expressed genes, including CXCLs and CCRs, were dramatically increased in Epi^CD147-OE^ mice compared to control mice by real-time polymerase chain reaction (PCR) (Fig. 1I, Supplementary Fig. 2), suggesting that CD147 might regulate the local inflammatory response through CXCLs or CCRs.

### Overexpression of CD147 in the epidermis facilitates DMBA/TPA-induced carcinogenesis

**Fig. 2:**
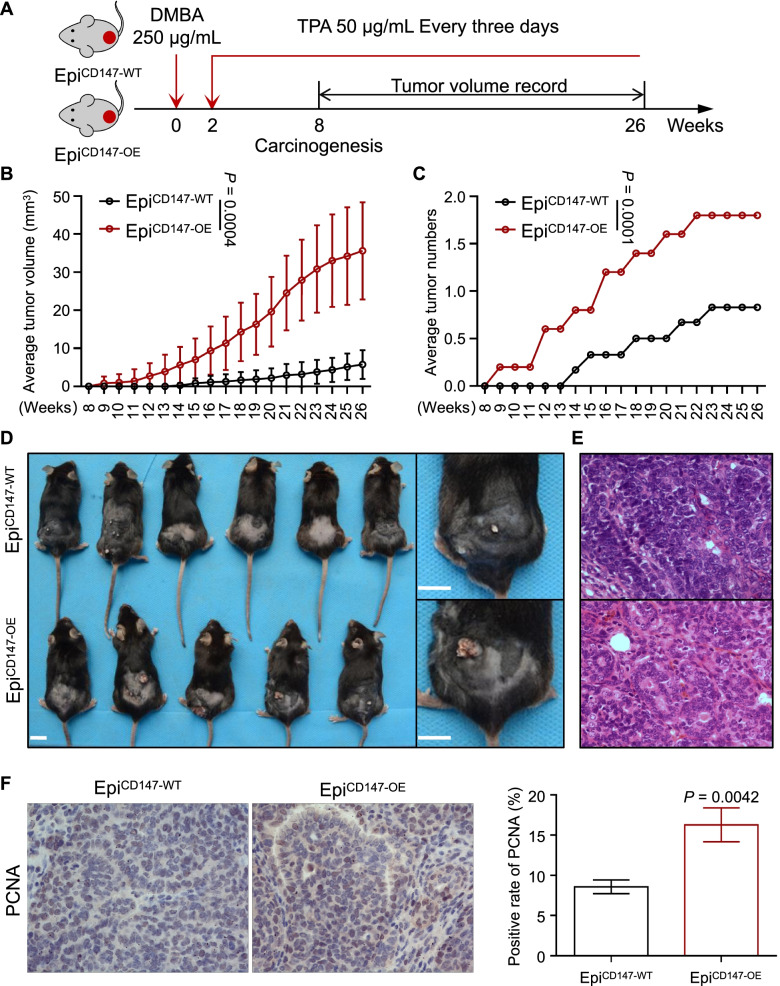
CD147 promotes the development of DMBA/TPA-induced skin carcinogenesis in transgenic mice. **A** Schematic diagram of the DMBA/TPA-induced skin carcinogenesis mouse model. Epi^CD147-OE^ mice and Epi^CD147-WT^ mice were treated with DMBA and TPA as described in the *Materials and Methods*. **B-C** Tumor volume (**B**) and number (**C**) were measured every week as described in the *Materials and Methods*. The tumor growth curves are shown as the mean tumor volume ± SD. The significance of differences was evaluated using one-way ANOVA. **D-E** Representative images of induced mouse models (**D**) and H&E staining of tumors (**E**) are shown at 26 weeks after stimulation. Scale bar = 1 cm. **F** CD147 promotes the proliferation of cSCC cells. Immunohistochemical staining of PCNA was performed as described in the *Materials and Methods*. Representative images were taken (**F left panel**), and bar chart graphs of the PCNA positive rate (%) (**F right panel**) are shown. Data are presented as the mean ± SD (*n* = 4). The significance of differences was evaluated using Student’s t-test

Given the spontaneous development of tumors in Epi^CD147-OE^ transgenic mice, we proposed that CD147 has an essential function in epidermal malignant transformation. To validate the role of CD147 in tumorigenesis, chemically induced tumorigenesis in Epi^CD147-OE^ mice with DMBA as the mutagen followed by TPA as the stimulus [20] was performed (Fig. 2A). Epi^CD147-OE^ mice (*n* = 6) initiated tumor formation after 9 weeks of TPA treatment, while tumors in the control mice (*n* = 7) did not develop until 14 weeks (Fig. 2B). The tumor size in Epi^CD147-OE^ mice was almost seven-fold larger, and the number of tumors was more than three-fold higher than that in control mice (Fig. 2B-E). Moreover, the number of tumor cells with positive PCNA staining in Epi^CD147-OE^ mice was almost two-fold greater than that in Epi^CD147-WT^ mice (Fig. 2F).

### Epidermal CD147 expression regulates the recruitment of MDSCs under inflammatory condition

**Fig. 3:**
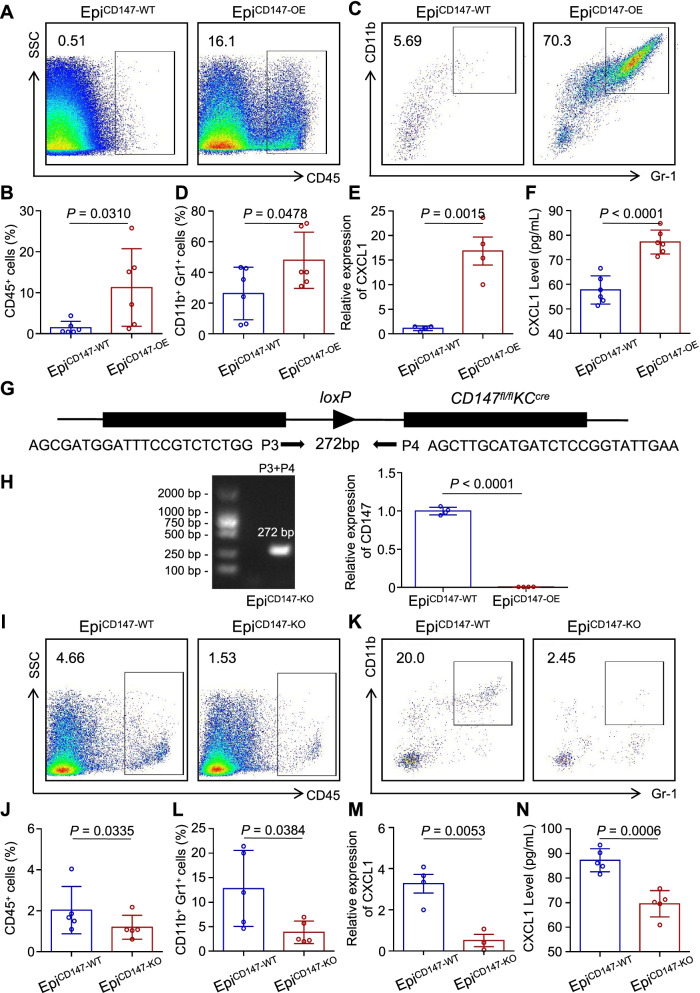
Epidermal CD147 regulates the recruitment of MDSCs under inflammation conditions. **A-B** The percentage of CD45^+^ cells is elevated in Epi^CD147-OE^ mice. Transgenic mice were treated with TPA for 12 h, and skin lesions were subjected to flow cytometry analysis*.* The gating strategy (**A**) and bar charts of the percentage of CD45^+^ cells (**B**) were presented. **C-D** The percentage of infiltrated CD11b^+^Gr1^+^ MDSCs was elevated in Epi^CD147-OE^ mice. The gating strategy (**C**) and bar charts of the percentage of CD11b^+^Gr1^+^ cells (**D**) were presented. **E-F** CXCL1 was increased in Epi^CD147-OE^ mice. RT-PCR (**E**) and ELISA (**F**) of CXCL1 were performed with TPA-treated skin lesion*.* **G-H** Schematic diagram of primers for genotyping and targeting strategies. Epi^CD147-KO^ genotyping was carried out with Primer 3 (P3) and Primer 4 (P4) (**G**). A 272 bp band was assessed for Epi^CD147-KO^ genotyping (**H left panel**), and DNA samples were prepared from total skin, and the expression of CD147 was test by q-PCR (**H right panel**). **I-J** The percentage of CD45^+^ cells was reduced in Epi^CD147-KO^ mice compared to Epi^CD147-WT^ mice. The gating strategy (**I**) and bar charts of the percentage of CD45^+^ cells (**J**) were presented. **K-L** The percentage of infiltrated CD11b^+^Gr1^+^ MDSCs was reduced in Epi^CD147-KO^ mice. The gating strategy (**K**) and bar charts of the percentage of CD11b^+^Gr1^+^ cells (**L**) were presented. **M-N** CXCL1 was decreased in Epi^CD147-KO^ mice compared to control mice. RT-PCR (**M**) and ELISA (**N**) of CXCL1 was performed with TPA-treated skin lesions*.* All data were presented as the mean ± SD. The significance of differences was evaluated using Student’s t-test

MDSCs has been well-documented as immunosuppressing cells that create immuno-suppressive microenvironment for tumorigenesis [21]. Given the critical role of MDSCs in tumor development, we utilized a transgenic mouse model for further investigation. TPA is a common chemical reagent used in multistage tumor formation procedures that acts through the induction of inflammation, cell proliferation and dedifferentiation. To study the effect of CD147 on TPA-induced inflammation, we exposed Epi^CD147-OE^ and Epi^CD147-WT^ mice to TPA for 12 hours, and then the skin lesions were subjected to flow cytometry. Interestingly, the proportion of inflammatory cells was significantly increased in Epi^CD147-OE^ mice (11.26 ± 3.87%) compared to Epi^CD147-WT^ mice (1.43 ± 0.63%) (Fig. 3A-B). Notably, we found that the percentage of CD11b^+^ Gr-1^+^ CD45^+^ cells, markers of MDSCs, in Epi^CD147-OE^ mice was dramatically elevated compared with that in Epi^CD147-WT^ mice (Fig. 3C-D).

It was well known that CXCLs recruit MDSCs to promote tumorigenesis [22]. Our previous results showed that CXCL1, 2 and 10 were remarkably upregulated in Epi^CD147-OE^ mice through RNA-seq and qRT-PCR, therefore, we assumed that elevated CXCLs contributed to MDSC infiltration in Epi^CD147-OE^ mice. As expected, the expression of CXCL1 in TPA-treated Epi^CD147-OE^ mouse skin lesions was almost 15-fold higher than that in Epi^CD147-WT^ mouse skin lesions (Fig. 3E), and subsequent ELISA results revealed that the expression of CXCL1 was significantly elevated in Epi^CD147-OE^ mouse skin lesions after TPA treatment (Fig. 3F). However, the alteration of CXCL2 and CXCL10 showed no significant difference between these two groups of mice (data not shown).

To better validate the role of CD147 in MDSC recruitment, we generated Epi^CD147-KO^ (KRT14Cre^+^/CD147^fl/fl^) transgenic mice (Fig. 3G-H) and exposed them to TPA treatment. As shown in Fig. 3I-L, CD147 deletion in the epidermis significantly blocked TPA-induced CD45^+^ cell infiltration and MDSC recruitment, indicating that epidermal expression of CD147 could affect MDSC infiltration. Moreover, the deletion of CD147 expression in the epidermis abrogated CXCL1 expression after treatment with TPA (Fig. 3M-N), indicating that CD147 regulates the recruitment of MDSCs through CXCL1.

### CD147 mediates the transformation of keratinocytes

**Fig. 4:**
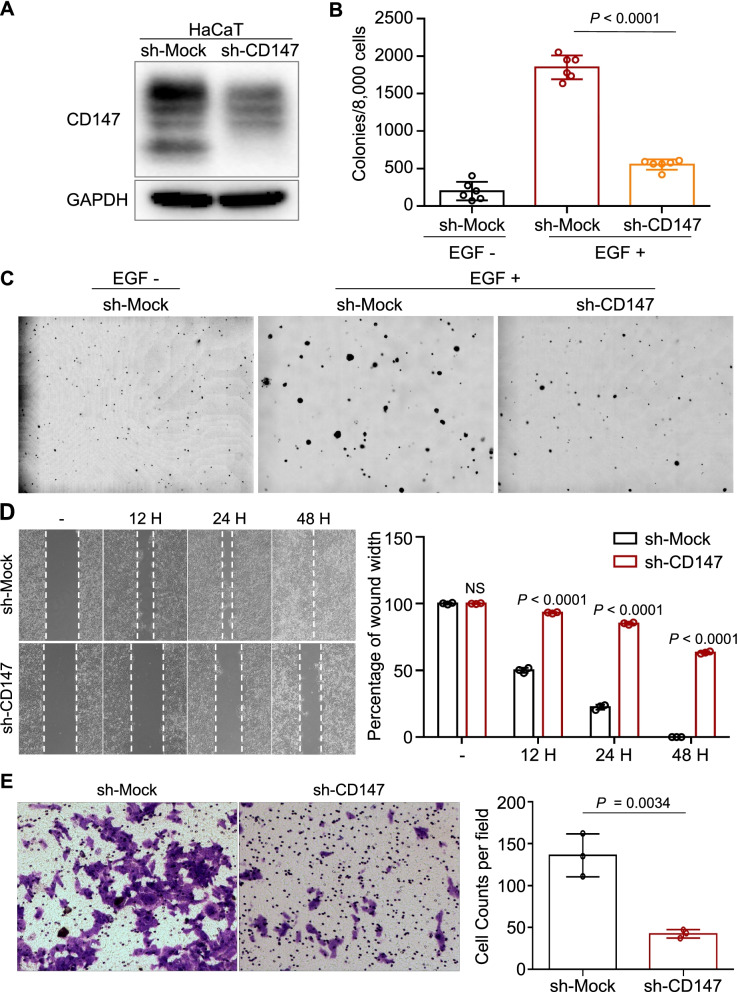
Knockdown of CD147 inhibits the malignant transformation of HaCaT cells. **A-C** Knockdown of CD147 suppresses the colony formation ability of HaCaT cells. HaCaT cells with stable knockdown of CD147 were generated by lentiviral infection. Protein was extracted from whole-cell lysates of HaCaT cells and subjected to immunoblot analysis using antibodies against CD147 as described in the *Materials and Methods*. GAPDH was used as a control (**A**). Cells were seeded into 6-well plates, and the number of colonies was counted as described in *Materials and Methods* (**B**). Representative images of HaCaT cells infected with sh-mock or sh-CD147 were presented (**C**). Data from multiple experiments are expressed as the mean ± SD. The significance of differences was evaluated using one-way ANOVA. **D-E** CD147-deficient HaCaT cells exhibit diminished migration and invasion abilities. A scratch assay was performed as described in the *Materials and Methods*. Representative images were taken at the indicated time points (**D left panel**), and bar chart graphs were shown from three independent experiments (**D right panel**). For the Transwell assay, the same number of cells (4 × 10^4^) was seeded into the upper layer of a chamber. The cells that migrated across the membrane were stained with crystal violet as described in the *Materials and Methods* (**E left panel**). Data represent the means (*n* = 4) ± SD of each group (**E right panel**). The significance of differences was evaluated using Student’s t-test

To further study the role of CD147 in transformation, we generated HaCaT cells with stable knockdown of CD147 expression (Fig. 4A), and an EGF-induced anchorage-independent cell growth assay was conducted to examine the effect of changes in CD147 expression on transformation. We found that colony formation was significantly reduced in CD147-knockdown HaCaT cells compared to control cells (Fig. 4B-C). Moreover, suppression of CD147 expression attenuated migration and invasion (Fig. 4D-E). Next, we over-expressed CD147 in CD147-knockdown HaCaT cells and performed functional studies including CCK-8, wound healing and transwell assay. The results showed that the proliferation, migration and invasion abilities of HaCaT cells were inhibited after knockdown of CD147, but were rescued after overexpression of CD147 (supplementary Fig. 3).

We also overexpressed CD147 in JB6 cells by lentiviral infection (Supplementary Fig. 4A), which showed that ectopic CD147 expression promoted the proliferation and colony formation of JB6 cells (Supplementary Fig. 4B-C) and increased their migration and invasion abilities (Supplementary Fig. 4D-F).

### CD147 promotes the malignant phenotype and recruits MDSCs in cSCC

**Fig. 5:**
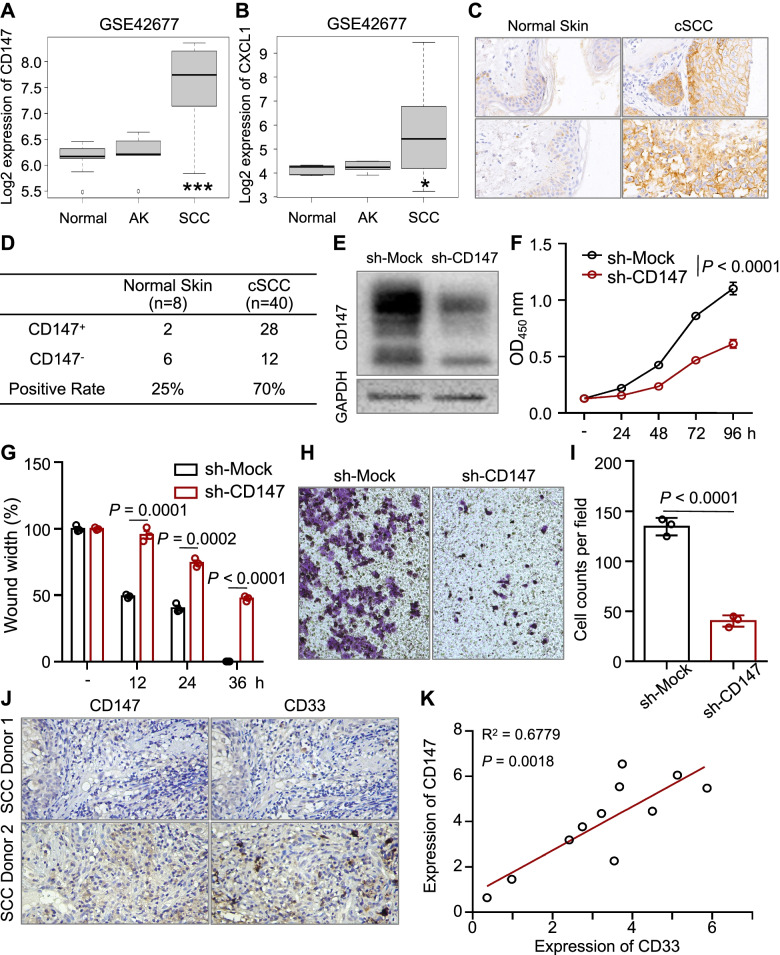
CD147 is overexpressed in cSCC and promotes the malignant biological behavior of A431 cells and relates with MDSCs. **A-B** The expression file of CD147 (**A**) and CXCL1 (**B**) in GSE42677. Box plots of mRNA expression levels for CD147 and CXCL1 in normal skin (*n* = 10), AK (*n* = 5), SCC (*n* = 10), based on GSE42677 data and plotted on a log2 scale (y-axis). **C-D** CD147 is highly expressed in cSCC tissues. Normal skin (*n* = 8) and cSCC (*n* = 40) tissues were collected, and immunohistochemistry staining of CD147 was performed as described in the *Materials and Methods.* Representative images were taken (**C**), and a summary graph of the CD147-positive rate was presented (**D**). **E-F** Knockdown of CD147 inhibits A431 cell growth. **E** Stable knockdown of CD147 in A431 cells was generated by lentiviral infection. Protein from whole-cell lysates of A431 cells was extracted and subjected to immunoblot analysis using antibodies against CD147 as described in the *Materials and Methods*. GAPDH was used as a loading control. **F** CD147-knocking down A431 cells showed a decreased growth rate. Cells were seeded into 96-well plates, and cell viability was examined by a CCK-8 kit. Data from multiple experiments are expressed as the mean ± SD. The significance of differences was evaluated using two-way ANOVA. **G-I** Inhibition of CD147 suppressed A431 cell migration and invasion. **G** The scratch assay was performed as described in the *Materials and Methods*. The bar chart graphs shown are from three independent experiments. Data are presented as the mean ± SD (*n* = 3). The significance of differences was evaluated using two-way ANOVA. **H** Transwell assays were performed as described in the *Materials and Methods*. **I** The number of invasive cells per field was calculated, and the data are presented as the mean ± SD (*n* = 4) of each group. The significance of differences between groups was evaluated by Student’s t-test. **J-K** The expression of CD147 and CD33 is positively correlated. The expression of CD147 and CD33 were detected in cSCC patients by immunohistochemistry staining as described in the *Materials and Methods.* Representative images were taken (**J**), and the correlation of CD147 and CD33 was determined using Pearson’s correlation analysis (**K**)

cSCCs occur via the malignant transformation of keratinocytes, therefore, we also investigated the role of CD147 in cSCC. The expression of CD147 and CXCL1 were found elevated in SCC patients compared normal control in dataset GSE42677 (Fig. 5A-B). Notably, the expression of CD147 showed the best correlation with CXCL1, but not with CXCL2, CXCL10 and IL6 (Supplementary Fig. 5), which is consistent with our results (Fig. 3F). We further detected the expression of CD147 in cSCC patients (*n* = 40), and found that the expression level CD147 was significantly higher than that in normal skin (*n* = 8) (*p* < 0.001) as shown in Fig. 5C-D. Next, we knocked down CD147 in the human cSCC cell line A431 by lentiviral infection (Fig. 5E). The growth of A431 cells was inhibited after knockdown of CD147 expression (Fig. 5F). Subsequent scratch and Transwell assays showed that inhibition of CD147 suppressed the migration and invasion of A431 cells (Fig. 5G-I).

As we found that MDSCs were increased in skin lesion of Epi^CD147-WT^ mice, hindering that those immunosuppressive cells might be altered in cSCC patients. Therefore, we tested the expression of CD147 as well as CD33 in cSCC tissues (*n* = 11) (Fig. 5 J). Interestingly, we noticed that the expression level of CD33 positive cells was positively related with level of CD147 expression (Fig. 5 K). This result further identifies the role of CD147 in regulating MDSCs.

### CD147 regulates CXCL1 expression through AP-1 via the RSK2 pathway

**Fig. 6:**
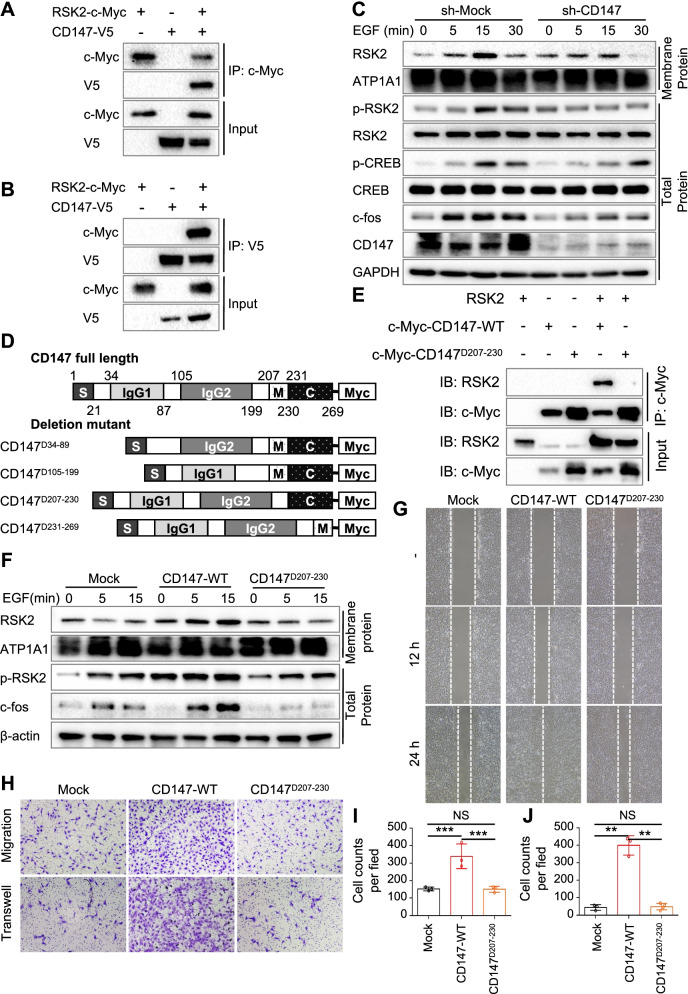
D207-230 silencing blocked the interaction of CD147 with RSK2 and inhibited cell growth and transformation. **A-B** RSK2 binds to CD147. 293 T cells were cotransfected with RSK2-c-Myc and CD147-V5 plasmids. Co-IP was performed with anti-c-Myc (**A**) or anti-V5 (**B**) antibodies, followed by immunoblotting with the indicated antibodies. **C** RSK2 is inhibited in CD147-knockdown keratinocytes. Membrane protein and whole-cell lysates of sh-Mock or sh-CD147 HaCaT cells stimulated with EGF were extracted and subjected to immunoblot analysis using indicated antibodies. ATP1A1 and GAPDH were used as loading control. **D-E** The CD147^D207-230^ mediates the interaction between CD147 and RSK2. Schematic diagram of truncated CD147 (**D**), and 293 T cells were cotransfected with RSK2 and CD147-c-Myc or CD147^D207-230^-c-Myc plasmids. Co-IP was performed with anti-c-Myc antibodies, followed by immunoblotting with the indicated antibodies (**E**). **F** CD147^D207-230^ attenuates EGF induced RSK2 signaling pathway. Membrane protein and whole-cell lysates of JB6-Mock, −CD147, and- CD147^D207-230^ cells stimulated with EGF were extracted and subjected to immunoblot analysis using indicated antibodies. ATP1A1 and GAPDH were used as loading control. **G-J** The CD147^D207-230^ blocks the pro-migration and -invasion ability in JB6 cells. JB6 transfected with Mock, CD147 and D207-230 were subjected to wound healing assay (**G**), migration (**H upper panel, I**) and transwell assay (**H lower panel, J**)

Our previous results found that p90 ribosomal S6 kinase 2 (RSK2) is a potential interacting partner of CD147 (data not shown). RSK2 is a member of RSK protein family, acting as a growth factor-stimulated protein kinase to mediating the MAPK pathway signal [23]. It is well documented that MAPK pathway is related to inflammatory pathway [24] and importantly, regulate CXCL1 expression [25]. Therefore, we inferred that CD147 may regulating CXCL1 expression by interacting with RSK2. To identify the interaction of CD147 and RSK2, we performed the co-Immunoprecipitation (Co-IP) by transfecting 293 T with RSK2 and CD147 and immunoprecipitated with anti-c-Myc antibody or anti-V5 antibody. Either CD147 or RSK2 was found in the corresponding immunoprecipitated complex (Fig. 6A-B), supporting that CD147 interacts with RSK2. RSK2 has two kinase domain for function. The C-terminal domain is activated by ERK-type MAP kinase and the N-terminal domain is activated by phosphoinositide-dependent kinase-1 (PDK1), which further triggers the phosphorylation and activation RSK2 [26]. Since the recruitment of membrane RSK2 represents the activation of RSK2, we stimulated HaCaT and HaCaT-CD147-knockout (CD147^KO^) cells with EGF and detected the expression of RSK2 and related proteins, to investigate the role of CD147 in RSK2 function. Interestingly, the expression of RSK2 in total protein did not show significantly change between Mock and CD147^KO^ cells. However, the level of RSK2 was decreased in membrane protein of CD147^KO^ cells, indicating that CD147 might regulate the membrane localization of RSK2. The level c-fos and p-CREB, the downstream molecules of RSK2, were also down-regulated in CD147^KO^ cells (Fig. 6C), suggesting that CD147 regulating RSK2 pathways. Correspondingly, the expression of membrane RSK2, c-fos and p-CREB were elevated after overexpressing CD147 in JB6 cells (CD147^OE^) (Supplementary Fig. 6A).

To better determine the association of CD147 and RSK2, we generated truncated CD147 in our lab [17], as shown in (Fig. 6D), and then, co-transfected RSK2 with mutant-CD147 constructed into 293 T cells. We found that the mutant of CD147^D207-230^ inhibited the association of CD147 with RSK2 (Supplementary Fig. 6B). To further confirm this result, 293 T cells were transfected with RSK2 and CD147 or CD147^D207-230^, and then, cell lystes were immunoprecipitated with anti-c-Myc antibody, as shown in Fig. 6E, RSK2 was not detected in the immunoprecipitated complex of CD147^D207-230^ group, suggesting the CD147 of D207-230 responses for CD147 bound to RSK2. Next, we therefore overexpressed CD147 and CD147^D207-230^ in JB6 cells and detected membrane RSK2 after stimulation of EGF. As expected, the elevation of membrane RSK2 were inhibited in CD147^D207-230^ (Fig. 6F). Since membrane RSK2 regulates cell biology, we detected the migration and invasion ability of JB6 cells after transfecting with CD147 or CD147^D207-230^. Interestingly, the CD147^D207-230^ did not promote JB6 cell migration and invasion, compared with CD147-WT (Fig. 6G-J). These results indicated that CD147^D207-230^ blocked the interaction of CD147 with RSK2 thus affecting cell growth and transformation.

**Fig. 7:**
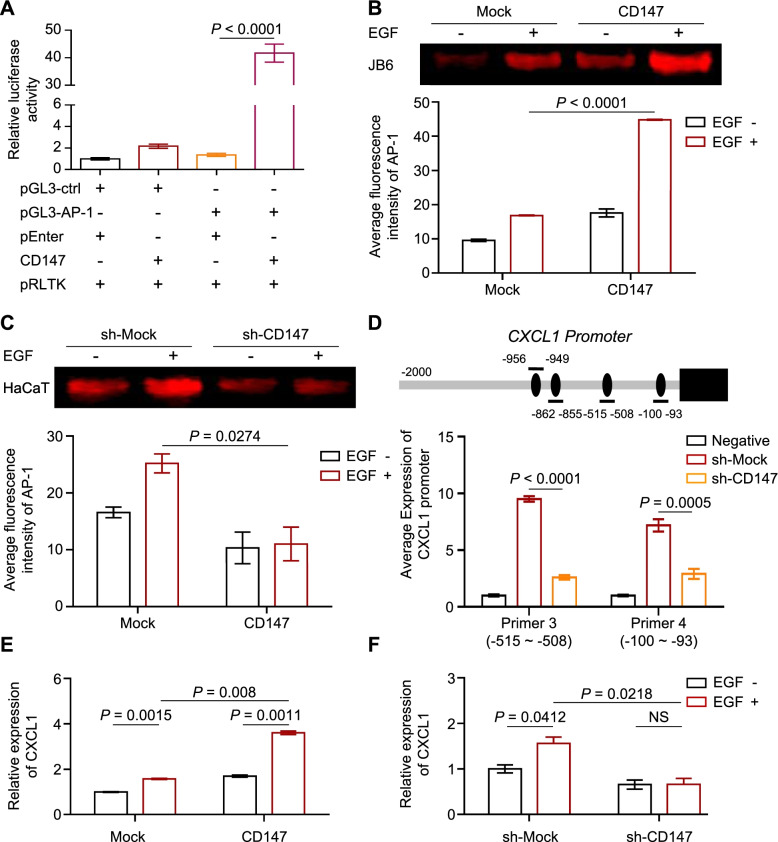
CD147 promotes the expression of CXCL1 by activating the transcriptional activity of AP-1. **A** Overexpression of CD147 increases AP-1 luciferase activity in keratinocytes. CD147 was transfected with pGL3-AP-1 or pGL3-ctrl plasmids containing the Renilla luciferase reporter gene, and the AP-1 activity was assessed by luciferase assay. Data from multiple experiments are expressed as the mean ± SD (*n* = 3). The significance of differences was evaluated using one-way ANOVA. **B-C** CD147 elevates AP-1 DNA binding activity in keratinocytes. Nuclear protein was extracted from CD147-overexpressing JB6 (**B upper panel**) and CD147-deficient HaCaT cells (**C upper panel**) treated with EGF and subjected to an EMSA. Bar charts of the average fluorescence intensity of AP-1 in JB6 (**B lower panel**) and HaCaT cells (**C lower panel**) were presented. Data were shown as the mean ± SD. The significance of differences was evaluated using two-way ANOVA. **D** CD147 enhances AP-1 association with CXCL1 promoter. Schematic diagram of the CXCL1 (**D upper panel**) promoters, and PROMO predicted several binding sites of AP-1. ChIP assays were performed to examine AP-1 recognition of the CXCL1 promoters (**D lower panel**). Data from multiple experiments are expressed as the mean ± SD (*n* = 3). The significance of differences was evaluated using Student’s t-test. The data of Primer 1 and Primer 2 were not shown as the experiment did not work. **E-F** The expression of CD147 correlates with the levels of CXCL1 in keratinocytes. CD147-overexpressing JB6 cells (**E**) and CD147-deficient HaCaT cells (**F**) were stimulated with EGF for 30 min and subjected to RT-PCR analysis of CXCL1. Data are presented as the mean ± SD. The significance of differences was evaluated using Student’s t-test

It is reported that suppression of RSK2 function inhibited AP-1 transactivation activity [27], and AP-1 is a well-known transcription factor that facilitates the progression of various cancers and mediates cell transformation [28]; therefore, we hypothesized that CD147 might regulate AP-1 activity. Plasmids encoding *AP-1*, the *Renilla* luciferase reporter gene and *CD147* were cotransfected into HEK293 cells. The results showed that *AP-1* promoter activity was dramatically increased in cells expressing CD147 (Fig. 7A). Next, we examined the effect of CD147 on AP-1 DNA binding activity through electrophoretic mobility-shift assays (EMSAs). As shown in Fig. 7B, ectopic CD147 expression increased AP-1 DNA binding activity, while knockdown of CD147 reduced its binding activity in the presence of EGF treatment (Fig. 7C). AP-1 plays a key transcriptional role in regulating the expression of a series of genes, including cytokines. Interestingly, we found potential AP-1-associated sites in CXCL1; therefore, we conducted a ChIP assay to examine whether AP-1 recognizes the CXCL1 promoters and to assess the effect of CD147 expression on AP-1 binding to its promoter. As expected, knockdown of CD147 attenuated the association of AP-1 with the CXCL1 promoters (Fig. 7D). Moreover, CXCL1 expression was up-regulated in ectopic CD147^OE^ JB6 cells, and inhibition of CD147 suppressed CXCL1 expression in HaCaT cells after EGF treatment (Fig. 7E-F).

### RSK2 inhibitor suppresses the tumor growth of cSCC through inhibiting MDSCs

**Fig. 8:**
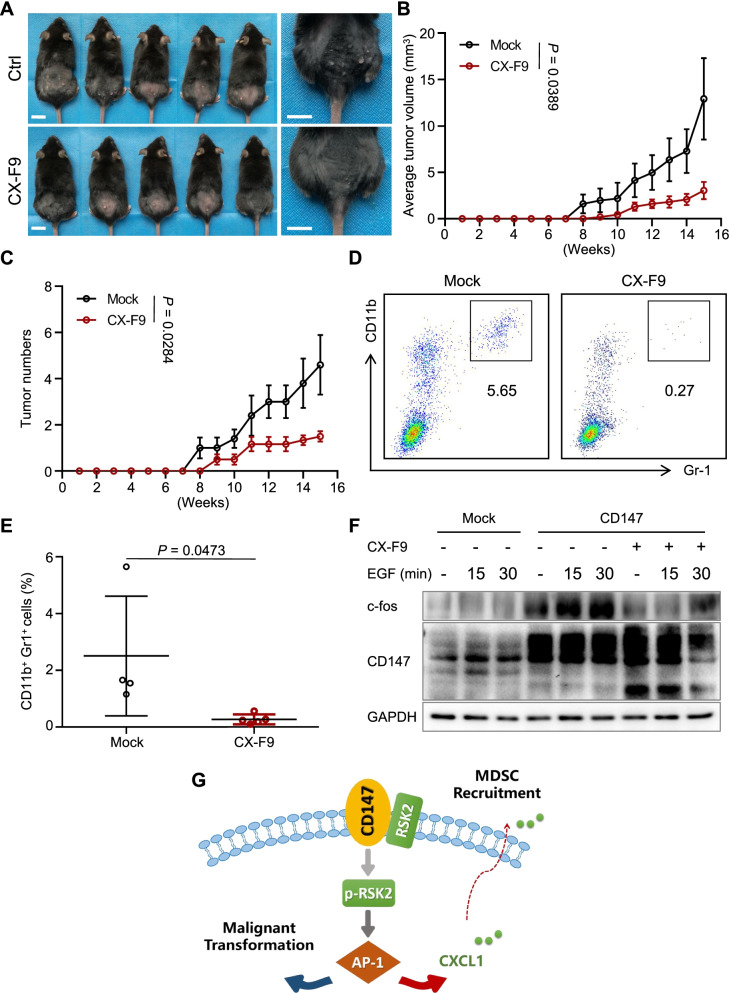
RSK2 inhibitor suppresses the DMBA/TPA-induced carcinogenesis through blocking MDSCs recruitment. **A** DMBA/TPA model was generated in Epi^CD147-OE^ mice and treated with CX-F9 by intraperitoneal injection twice a week. Representative images of induced mouse models are shown at 16 weeks after stimulation. Scale bar = 1 cm. **B-C** Tumor volume (**B**) and numbers (**C**) were measured every week as described in the *Materials and Methods*. The tumor growth curves are shown as the mean tumor volume ± SD. The significance of differences was evaluated using one-way ANOVA. **D-E** The percentage of CD11b^+^Gr1^+^ MDSCs is reduced in CX-F9 treated mice. Skin lesions were subjected to flow cytometry as described in the *Materials and Methods.* The gating strategy (**D**) and bar charts of the percentage of CD11b^+^Gr1^+^ MDSCs (**E**) are shown. Data are presented as the mean ± SD. The significance of differences was evaluated using Student’s t-test. **F** CX-F9 inhibits c-fos expression in JB6 cells. Whole-cell lysates of JB6-Mock or JB6-CD147 cells stimulated with EGF and treated with/without CX-F9 were extracted and subjected to immunoblot analysis using indicated antibodies. GAPDH was used as loading control. **G** Schematic diagram of the role of CD147 in the malignant transformation of keratinocytes

We previously found that CX-F9 was a RSK2 inhibitor and suppressed the development of melanoma [29]. Therefore, CX-F9 may exert a therapeutic function in cSCC by inhibiting RSK2. We then generated the DMBA/TPA skin carcinogenesis model in Epi^CD147-OE^ mice and then treated with CX-F9 twice a week by intraperitoneal injection (i.p). Surprisingly, we found the tumor numbers and volumes were inhibited after CX-F9 treatment (Fig. 8A-C). Moreover, CX-F9 significantly decreased the MDSCs in skin lesion of DMBA/TPA model (Fig. 8D-E), suggesting CX-F9 is a potential promising inhibitor for cSCC therapy. Given the above results already approved that CD147 activates the AP-1 through RSK2, we examined the effect of CX-F9 on the activation of AP-1. JB6-CD147^OE^ cells were treated with 10 μM CX-F9, and then, stimulated with 100 ng/mL EGF and the results showed the c-fos was elevated in CD147-overexpressing JB6 cells compared to control cells. However, it was inhibited after treatment of CX-F9, indicating that CX-F9 inhibits AP-1 activation induced by CD147 overexpression (Fig. 8F).

Taken together, our results reveal that CD147 activates the AP-1 pathway through RSK2, which facilitates epidermal transformation and increases the expression of CXCL1 to recruit MDSCs, eventually promoting tumorigenesis (Fig. 8G).

## Discussion

CD147 is well known to be involved in the invasion and metastasis of cancer cells [16, 30]. The role of CD147 in the malignant transformation of normal cells and the crosstalk between keratinocytes and immune cells remain unclear. In this study, our findings showed that approximately 55% of Epi^CD147-OE^ mice spontaneously developed tumors at 12 months, indicating that CD147 facilitates malignant transformation. Crosstalk between resident keratinocytes and immune cells, including T cells, macrophages and MDSCs, plays essential roles during epidermal transformation and tumorigenesis. Keratinocytes can be activated by a variety of cytokines secreted by immune cells, such as interferon-γ (IFN-γ), through STAT1 [31], and interleukin-22 (IL-22) through STAT3 [32, 33]. In addition, activated keratinocytes also produce various inflammatory factors, such as TNF-a, IL-6, CXCL1, and CXCL2, which induce the recruitment of immune cells, including MDSCs and macrophages, and facilitate malignant transformation [34, 35]. Evidence has demonstrated that CXCL1 plays key roles in the generation and recruitment of MDSCs during carcinogenesis [36, 37].

As key immunosuppressive cells, MDSCs are abundant in cancers and inhibit the function of natural killer (NK) cells, B cells, and T cells. In addition, MDSCs regulate the differentiation of dendritic cells and the polarization of M2 macrophages, hence supporting tumor cell escape from immune surveillance [10]. By blocking peptide-MHC binding with CD8^+^ T cells, MDSCs disrupt the T cell response to tumor-specific antigens [38]. In addition to interfering with the polarization of CD8^+^ T cells [39], MDSCs are a major source of TGF-β, which significantly inhibits the expression of cytotoxic proteins such as perforin and granzyme B in cytotoxic T lymphocytes (CTLs) [40]. In squamous cell carcinoma, MDSCs promote the cancer stem cell phenotype via the epithelial-mesenchymal transition (EMT) pathway [41]. They also inhibit the proliferation of T cells and increase angiogenesis via cyclooxygenase (COX)-2 [42]. Moreover, an increase in Th17 cells induced by MDSCs together with a decrease in T cells leads to a chronic inflammatory microenvironment that eventually facilitates tumor formation [43].

RSK2 is reported to be a key regulator in human skin cancer as a serine/threonine kinase. It plays various roles in cell biology, including cell transformation [44] and differentiation [45]. Knocking down of RSK2 inhibited the EGF-induced anchorage-independent transformation of HaCaT cells, indicating the important function of RSK2 in epidermal cell transformation [46]. As a critical downstream member in MAKP pathway, RSK2 is found to regulate the inflammation signal through TRAF6 [47]. Our previous study showed that CD147/TRAF6 regulates EGF-induced cell transformation and cSCC malignant phenotype [19]. Considering these, the CD147-interating protein, RSK2, might be important in the CD147 mediated epidermal cell transformation. Therefore, we identified the interaction of CD147 and RSK2. Importantly, the membrane RSK2 activates signaling pathways that enhance cell growth and transformation. The recruitment of PDK1 to RSK2 activates the N-terminal kinase of RSK2, which further triggers the phosphorylation and activation RSK2. The activation of RSK2 is critical in the cell transformation induced by tumor promoters such as EGF and TPA [48]. Our results found that membrane RSK2 was elevated in CD147^OE^ cells, suggesting the activation of RSK2 pathway.

AP-1 is documented as a RSK2-induced downstream pathway [27] that triggers cell transformation and promotes the development of tumors, including cSCC [49, 50]. Especially, AP-1 regulates the cell transformation induced by EGF or TPA [51]. As a transcription factor, AP-1 is also involved in the production of inflammatory cytokines such as IL-6 [52, 53] and TNFα, which supports the malignant phenotype of cancer cells [54]. Here, our findings showed that overexpression of CD147 increased AP-1 DNA binding activity and transcriptional activity; moreover, CXCL1 was identified as targets of AP-1, and suppression of CD147 expression inhibited CXCL1 expression. AP-1 was shown to associate with the CXCL1 promoters, suggesting that CD147 regulates CXCL1 expression through the AP-1 transcriptional factor.

We previously identified a RSK2 inhibitor, CX-F9, inhibited the development of melanoma cells [29]. In this study, we treated DMBA/TPA-induced skin tumor mice model with CX-F9 to investigate the therapeutic potential of RSK2 in cSCC. We found that CX-F9 notably inhibited the growth of DMBA/TPA-induced tumors and the percentage of MDSCs in skin lesions.

## Conclusion

With this study, we demonstrate for the first time that CD147 promotes the malignant transformation of keratinocytes and the tumorigenesis of skin cancers. In addition to enhancing the malignant biological behaviors of normal cells, CD147 recruits MDSCs into the epidermis, thereby forming a protumor microenvironment through CXCL1 via RSK2. CX-F9 could inhibit this process by inhibiting RSK2. Taken together, our work reveals a novel mechanism of the cell transformation and onset of skin cancers and indicates that targeting RSK2 is a promising treatment strategy for skin cancers.

## Supplementary Information

**Additional file 1: Supplementary Figure 1.** RNA Sequencing Profile of Epi^CD147-OE^ Mice. (A) The top 20 positively enriched KEGG pathways are shown in the bubble chart. The x-axis is the enrichment score, and the y-axis is the enriched pathways. (B) CXCLs are node proteins in the PPI analysis of Epi^CD147-OE^ mice. The PPI network was analyzed using STRING online (https://string-db.org/).

**Additional file 2: Supplementary Figure 2.** RT-PCR Analyses of the Effect of CD147 on the Gene Expression Profile of Epi^CD147-OE^ Mice. RNA was extracted from Epi^CD147-OE^ and Epi^CD147-WT^ mice. RT-PCR was then performed with different primers (MMP8, CCL1, IL-1β, CCL2, CCL3 and CCL4) as described in the *Materials and Methods*. The data from multiple experiments (*n* = 4) are expressed as the mean ± SD. The significance of differences was evaluated using Student’s t-test.

**Additional file 3: Supplementary Figure 3.** Overexpression of CD147 in CD147-knockdown HaCaT cells rescued the phenotype. (A) Overexpression of CD147 promote the growth of HaCaT cells with CD147-knockdown. Stable overexpression of CD147 in JB6 cells was generated by lentiviral infection. Cells were seeded into 96-well plates, and cell viability was examined by a CCK-8 kit as described in the *Materials and Methods*. Data from multiple experiments are expressed as the mean ± SD. The significance of differences was evaluated using two-way ANOVA. (B-E) Overexpression of CD147 promotes the migration and invasion abilities of CD147-knockdown HaCaT cells. The scratch assay was performed as described in the *Materials and Methods* (B). The bar chart graphs shown are from three independent experiments (C). Data are presented as the mean ± SD (*n* = 3). The significance of differences was evaluated using two-way ANOVA. Transwell assays were performed as described in the *Materials and Methods* (D). The number of invasive cells per field was calculated, and the data are presented as the mean ± SD (*n* = 4) of each group (E). The significance of differences between cells was evaluated by one-way ANOVA.

**Additional file 4: Supplementary Figure 4.** Overexpression of CD147 promotes the malignant transformation of JB6. (A-B) Overexpression of CD147 accelerates the growth of JB6 in vitro. Stable overexpression of CD147 in JB6 cells was generated by lentiviral infection. Whole-cell lysates of JB6 cells were extracted and subjected to immunoblot analysis using antibodies against CD147 as described in the *Materials and Methods*. GAPDH was used as a control (A). CD147-overexpressing JB6 cells showed an increased growth rate (B). Cells were seeded into 96-well plates, and cell viability was examined by a CCK-8 kit as described in the *Materials and Methods*. Data from multiple experiments are expressed as the mean ± SD. The significance of differences was evaluated using two-way ANOVA. (C) CD147 increases the colony formation ability of JB6 cells. Cells were seeded into 6-well plates, and the number of foci was counted as described in the *Materials and Methods*. Data from three independent experiments are expressed as the mean ± SD. The significance of differences was evaluated using Student’s t-test. (D-F) Upregulation of CD147 promotes the migration and invasion abilities of JB6 cells. The scratch assay was performed as described in the *Materials and Methods*. The bar chart graphs shown are from three independent experiments (D). Data are presented as the mean ± SD (*n* = 3). The significance of differences was evaluated using two-way ANOVA. Transwell assays were performed as described in the *Materials and Methods* (E). The number of invasive cells per field was calculated, and the data are presented as the mean ± SD (*n* = 4) of each group (F). The significance of differences between cells was evaluated by Student’s t-test.

**Additional file 5: Supplementary Figure 5.** Expression of CD147 related genes in normal skin, actinic keratosis, and cSCC in datasets of GSE42677. The correlation of CD147 with CXCL1 (A), CXCL2 (B), CXCL10 (C) and IL6 (D) were determined using Pearson’s correlation analysis.

**Additional file 6: Supplementary Figure 6.** Overexpression of CD147 promotes the activation of RSK2 and the interaction of CD147 and RSK2 is mediated by CD147^D207-230^. (A) Whole-cell lysates of JB6-Mock or JB6-CD147^OE^ cells stimulated with EGF were extracted and subjected to immunoblot analysis using indicated antibodies. GAPDH was used as a control. (B) The CD147^D207-230^ mediates the interaction between CD147 and RSK2. RSK2 and truncated CD147 were co-transfected into 293 T cells. Co-IP was performed with anti-c-Myc antibodies, followed by immunoblotting with the indicated antibodies.

## Data Availability

The datasets used and/or analysed during the current study are available from the corresponding author on reasonable request.

## Abbreviations

cSCC: Cutaneous squamous cell carcinoma
MDSCs: Myeloid-derived suppressor cells
TME: Tumor microenvironment
DCs: Dendritic cells
NO: Nitric oxide
ROS: Reactive oxygen species
Epi^CD147-OE^: Transgenic mice with epidermal overexpressing of CD147
Epi^CD147-KO^: Transgenic mice with epidermal knockout of CD147
DMBA/TPA: 7,12-dimethylbenzanthracene/12-O-tetradecanoylphorbol-13-acetate
GSEA: Gene set enrichment analysis
KEGG: Encyclopedia of Genes and Genomes
PPI: Protein-protein interaction
EMSAs: Electrophoretic mobility-shift assays
IFN-γ: Interferon-γ
IL: Interleukin
NK: Natural killer cells
CTLs: Cytotoxic T lymphocytes
COX: Cyclooxygenase
