# Supplementary figures and images for: CD147 mediates epidermal malignant transformation through the RSK2/AP-1 pathway
Source: J Exp Clin Cancer Res. 2022 Aug 13;41:246. doi: 10.1186/s13046-022-02427-w (PMC9375950; doi:10.1186/s13046-022-02427-w)

## sFigure 1

**A**

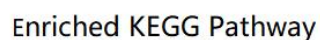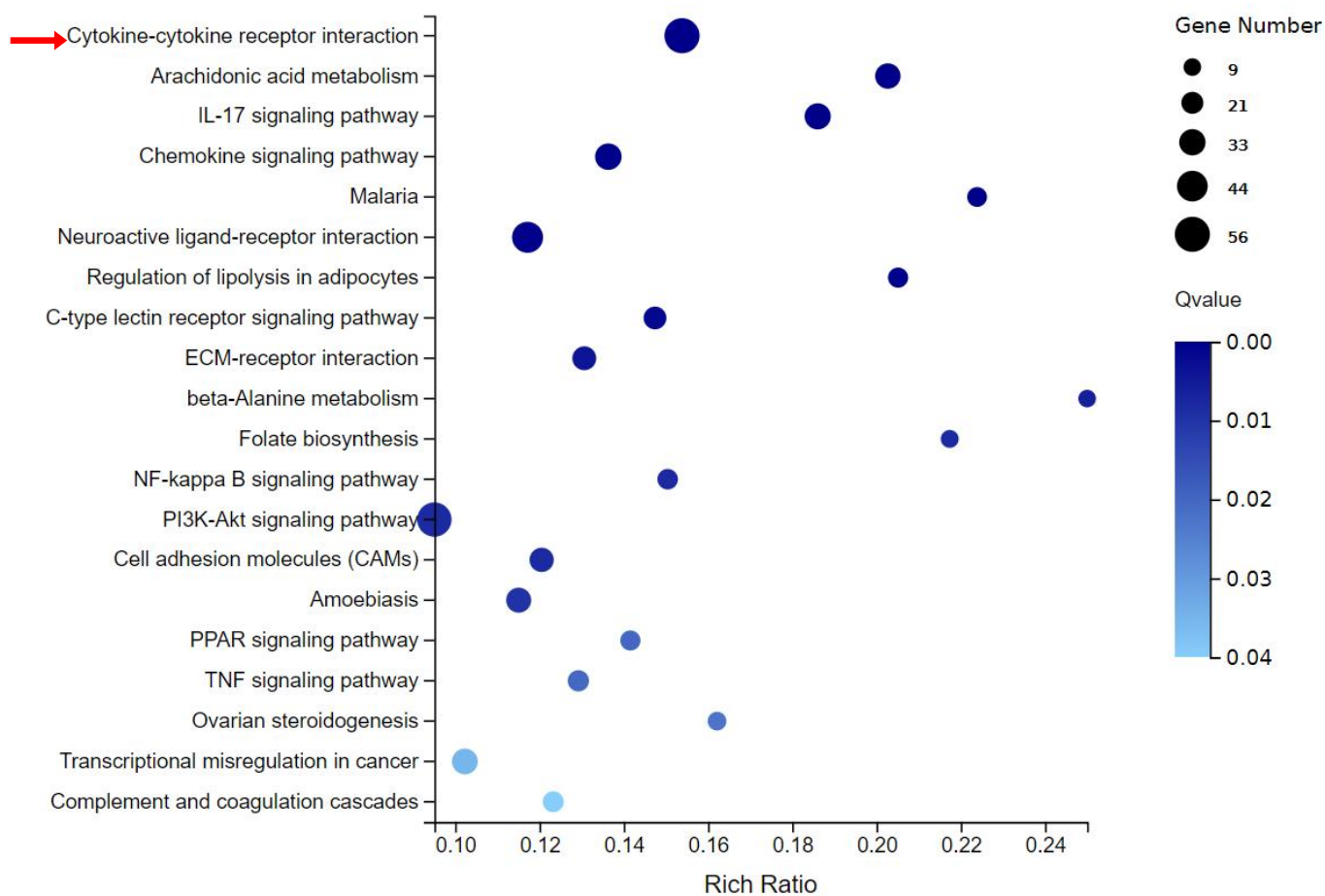

# B

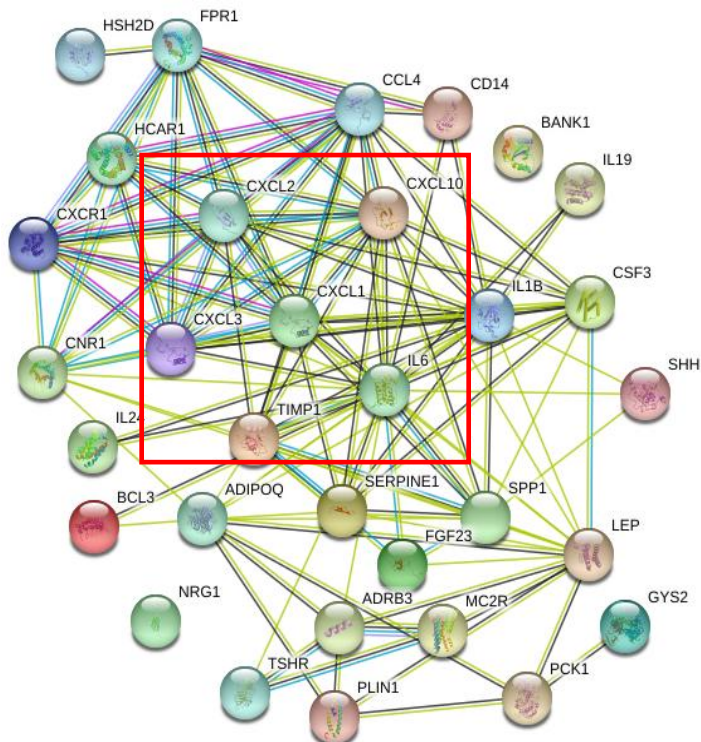

Supplement: Supplementary file 1 — Additional file 1: Supplementary Figure 1. RNA Sequencing Profile of EpiCD147-OE Mice. (A) The top 20 positively enriched KEGG pathways are shown in the bubble chart. The x-axis is the enrichment score, and the y-axis is the enriched pathways. (B) CXCLs are node proteins in the PPI analysis of EpiCD147-OE mice. The PPI network was analyzed using STRING online (https://string-db.org/). [file 13046_2022_2427_MOESM1_ESM.pdf]

**sFigure 2**

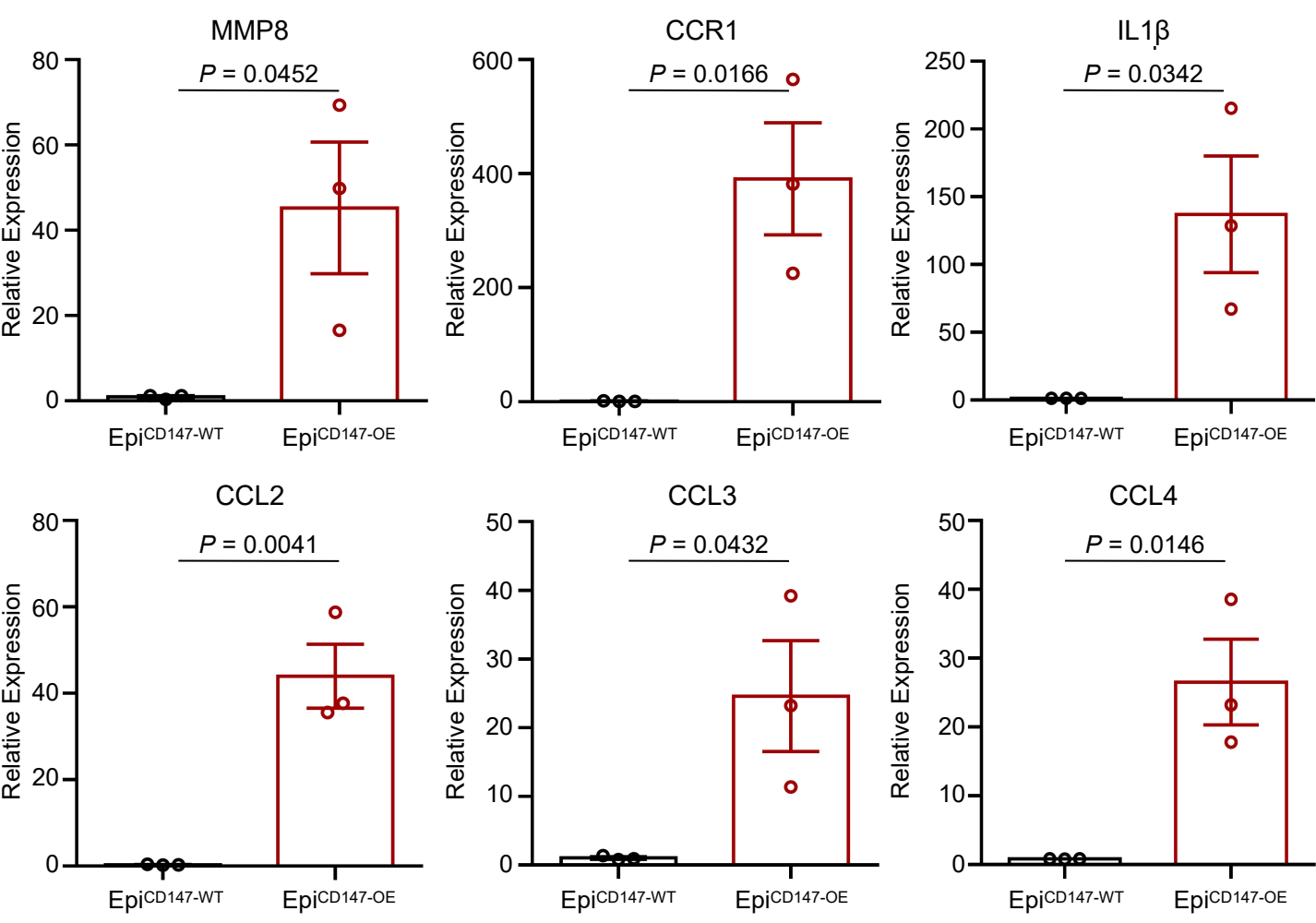

Supplement: Supplementary file 2 — Additional file 2: Supplementary Figure 2. RT-PCR Analyses of the Effect of CD147 on the Gene Expression Profile of EpiCD147-OE Mice. RNA was extracted from EpiCD147-OE and EpiCD147-WT mice. RT-PCR was then performed with different primers (MMP8, CCL1, IL-1β, CCL2, CCL3 and CCL4) as described in the Materials and Methods. The data from multiple experiments (n = 4) are expressed as the mean ± SD. The significance of differences was evaluated using Student’s t-test. [file 13046_2022_2427_MOESM2_ESM.pdf]

**sFigure 3**

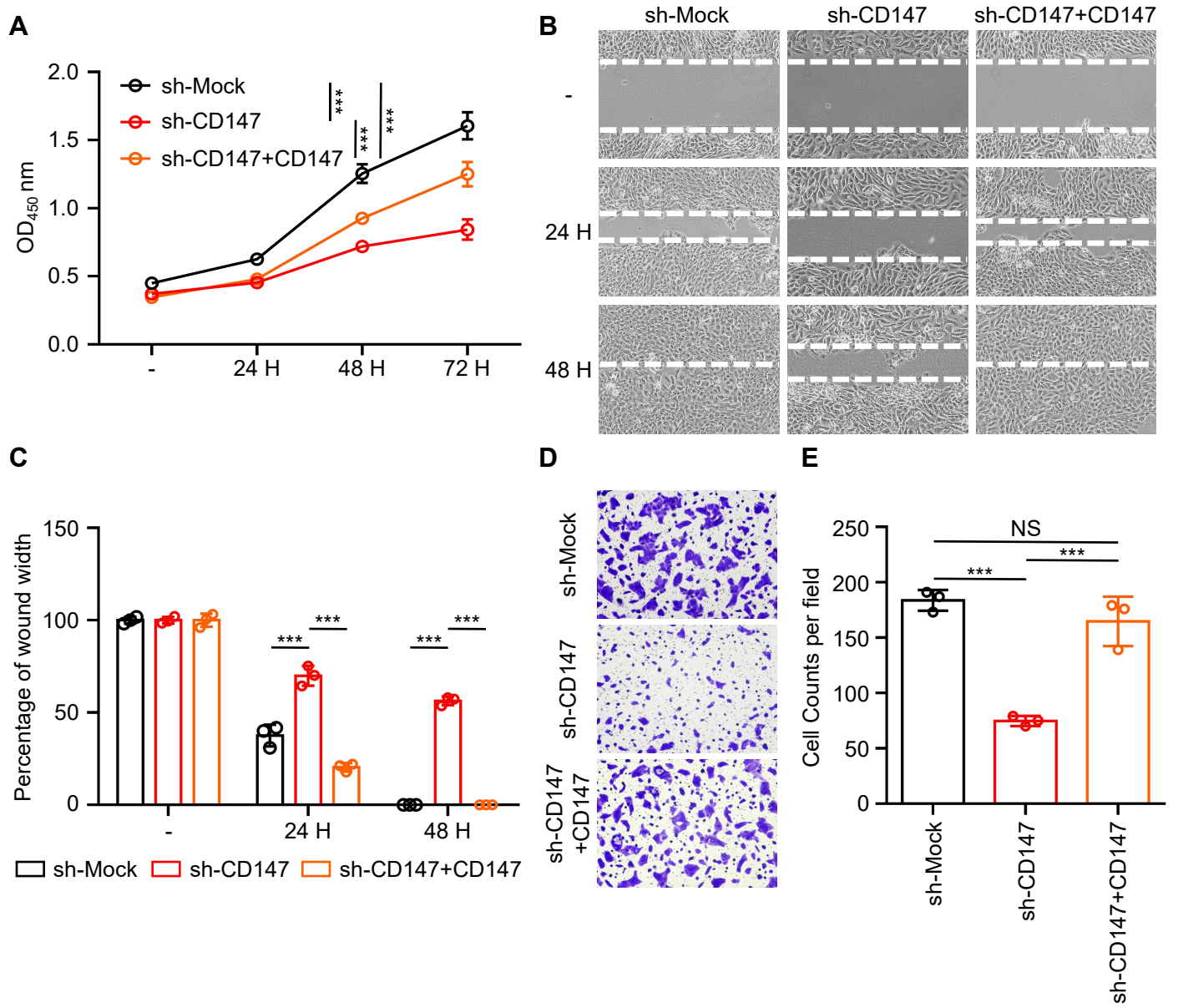

Supplement: Supplementary file 3 — Additional file 3: Supplementary Figure 3. Overexpression of CD147 in CD147-knockdown HaCaT cells rescued the phenotype. (A) Overexpression of CD147 promote the growth of HaCaT cells with CD147-knockdown. Stable overexpression of CD147 in JB6 cells was generated by lentiviral infection. Cells were seeded into 96-well plates, and cell viability was examined by a CCK-8 kit as described in the Materials and Methods. Data from multiple experiments are expressed as the mean ± SD. The significance of differences was evaluated using two-way ANOVA. (B-E) Overexpression of CD147 promotes the migration and invasion abilities of CD147-knockdown HaCaT cells. The scratch assay was performed as described in the Materials and Methods (B). The bar chart graphs shown are from three independent experiments (C). Data are presented as the mean ± SD (n = 3). The significance of differences was evaluated using two-way ANOVA. Transwell assays were performed as described in the Materials and Methods (D). The number of invasive cells per field was calculated, and the data are presented as the mean ± SD (n = 4) of each group (E). The significance of differences between cells was evaluated by one-way ANOVA. [file 13046_2022_2427_MOESM3_ESM.pdf]

**sFigure 4**

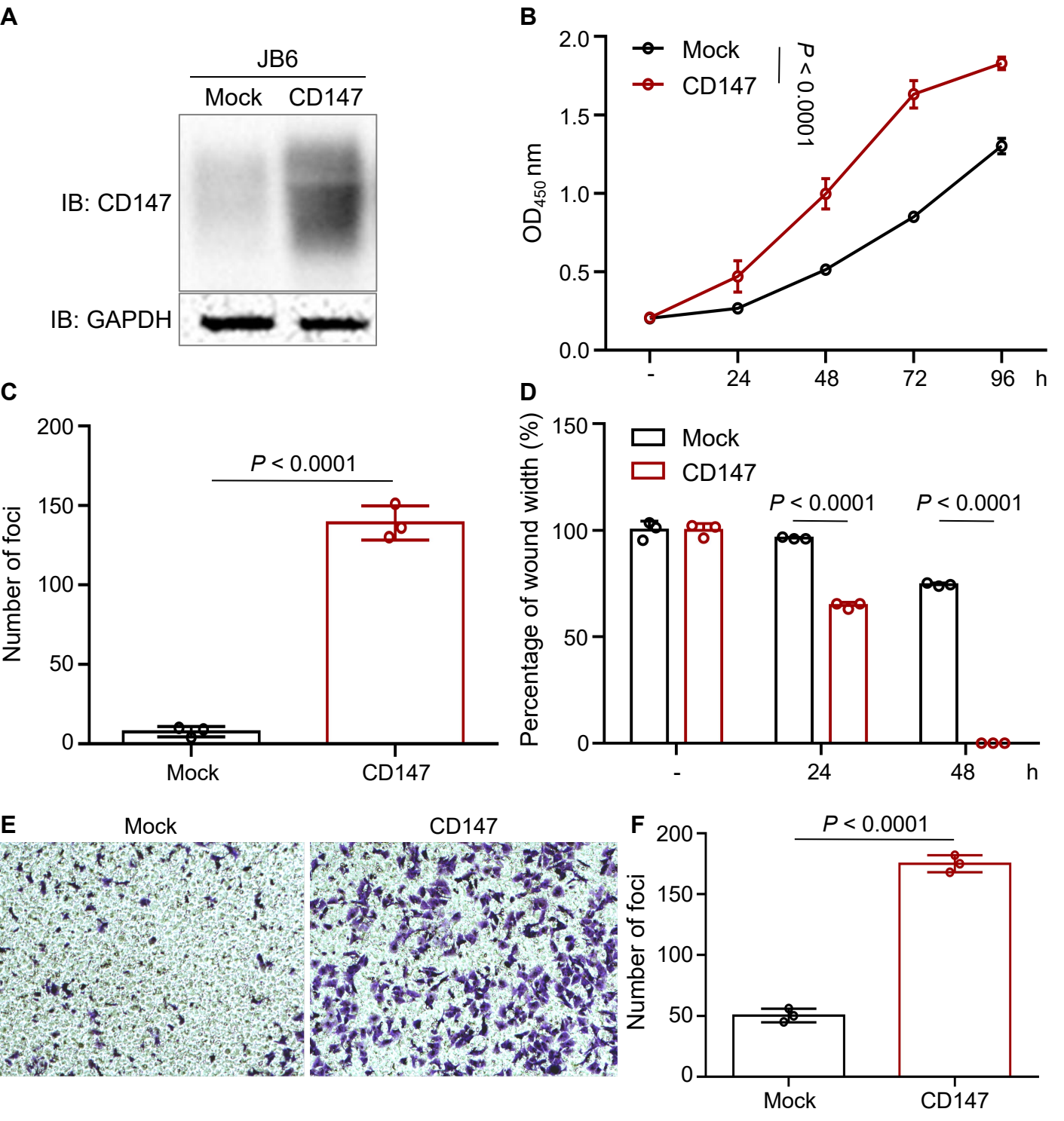

Supplement: Supplementary file 4 — Additional file 4: Supplementary Figure 4. Overexpression of CD147 promotes the malignant transformation of JB6. (A-B) Overexpression of CD147 accelerates the growth of JB6 in vitro. Stable overexpression of CD147 in JB6 cells was generated by lentiviral infection. Whole-cell lysates of JB6 cells were extracted and subjected to immunoblot analysis using antibodies against CD147 as described in the Materials and Methods. GAPDH was used as a control (A). CD147-overexpressing JB6 cells showed an increased growth rate (B). Cells were seeded into 96-well plates, and cell viability was examined by a CCK-8 kit as described in the Materials and Methods. Data from multiple experiments are expressed as the mean ± SD. The significance of differences was evaluated using two-way ANOVA. (C) CD147 increases the colony formation ability of JB6 cells. Cells were seeded into 6-well plates, and the number of foci was counted as described in the Materials and Methods. Data from three independent experiments are expressed as the mean ± SD. The significance of differences was evaluated using Student’s t-test. (D-F) Upregulation of CD147 promotes the migration and invasion abilities of JB6 cells. The scratch assay was performed as described in the Materials and Methods. The bar chart graphs shown are from three independent experiments (D). Data are presented as the mean ± SD (n = 3). The significance of differences was evaluated using two-way ANOVA. Transwell assays were performed as described in the Materials and Methods (E). The number of invasive cells per field was calculated, and the data are presented as the mean ± SD (n = 4) of each group (F). The significance of differences between cells was evaluated by Student’s t-test. [file 13046_2022_2427_MOESM4_ESM.pdf]

**sFigure 5**

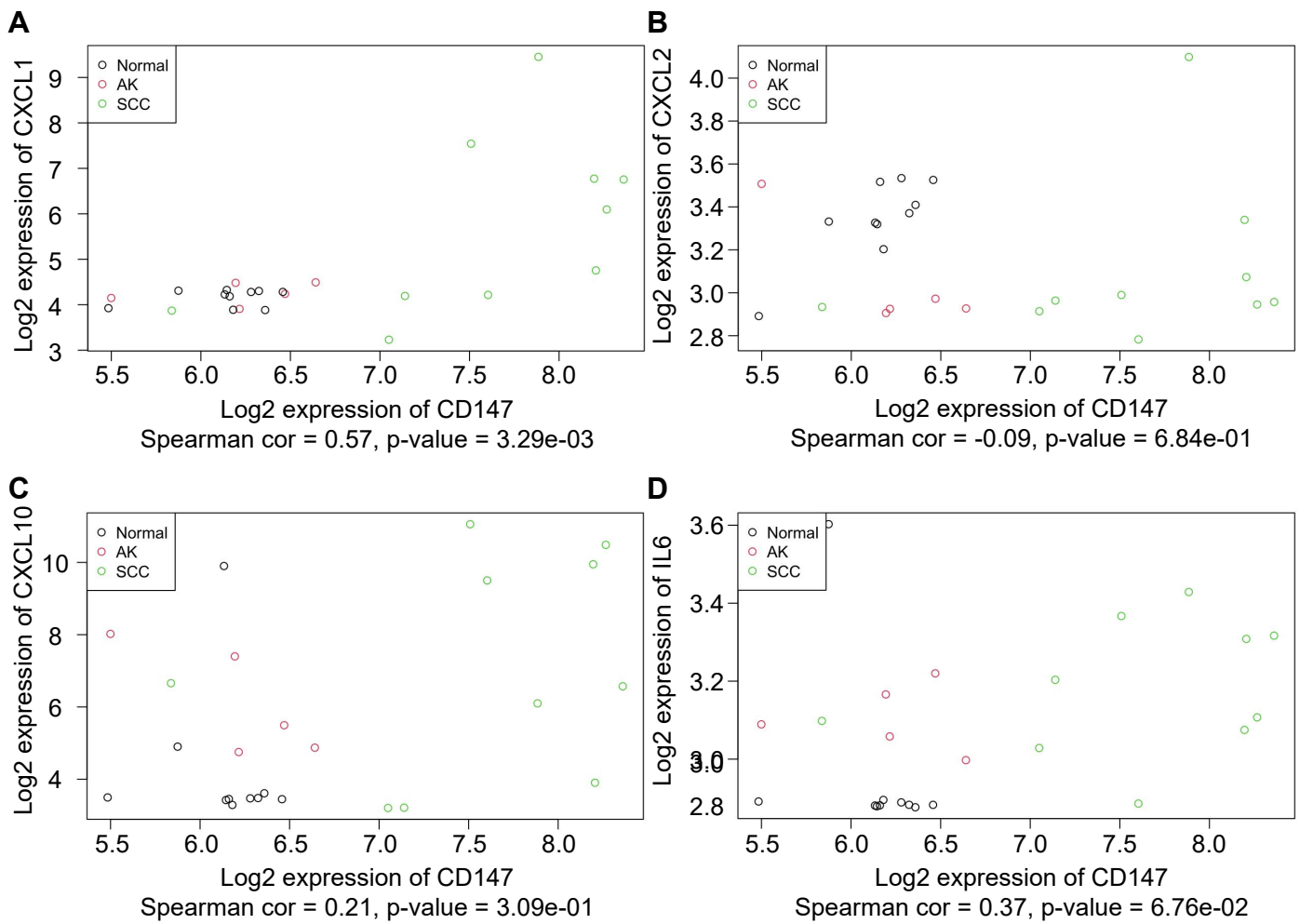

Supplement: Supplementary file 5 — Additional file 5: Supplementary Figure 5. Expression of CD147 related genes in normal skin, actinic keratosis, and cSCC in datasets of GSE42677. The correlation of CD147 with CXCL1 (A), CXCL2 (B), CXCL10 (C) and IL6 (D) were determined using Pearson’s correlation analysis. [file 13046_2022_2427_MOESM5_ESM.pdf]

**sFigure 6**

**A**

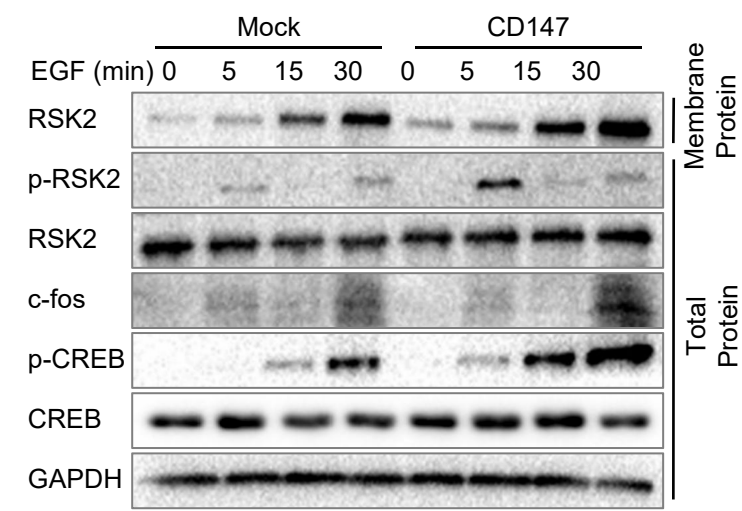

**B**

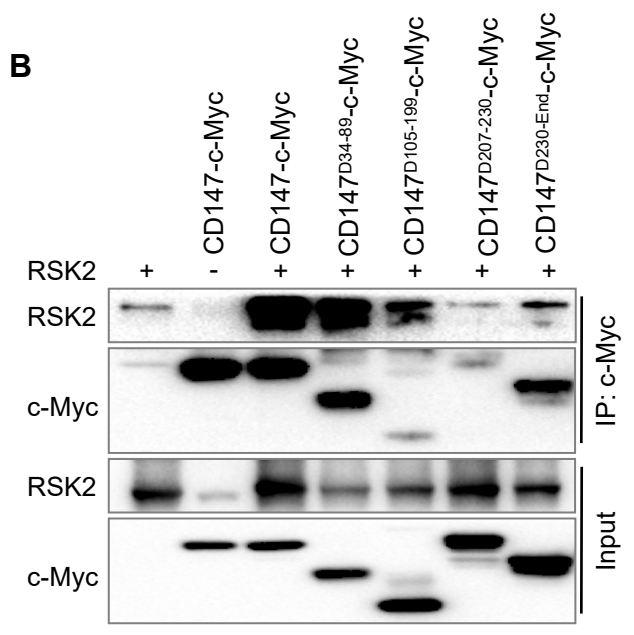

Supplement: Supplementary file 6 — Additional file 6: Supplementary Figure 6. Overexpression of CD147 promotes the activation of RSK2 and the interaction of CD147 and RSK2 is mediated by CD147D207-230. (A) Whole-cell lysates of JB6-Mock or JB6-CD147OE cells stimulated with EGF were extracted and subjected to immunoblot analysis using indicated antibodies. GAPDH was used as a control. (B) The CD147D207-230 mediates the interaction between CD147 and RSK2. RSK2 and truncated CD147 were co-transfected into 293 T cells. Co-IP was performed with anti-c-Myc antibodies, followed by immunoblotting with the indicated antibodies. [file 13046_2022_2427_MOESM6_ESM.pdf]
